# A Review of Heterogeneous Photocatalysis for Water and Surface Disinfection

**DOI:** 10.3390/molecules20045574

**Published:** 2015-03-30

**Authors:** John Anthony Byrne, Patrick Stuart Morris Dunlop, Jeremy William John Hamilton, Pilar Fernández-Ibáñez, Inmaculada Polo-López, Preetam Kumar Sharma, Ashlene Sarah Margaret Vennard

**Affiliations:** 1 Nanotechnology and Integrated BioEngineering Centre, Ulster University, Newtownabbey, Northern Ireland BT37 0QB, UK; E-Mails: psm.dunlop@ulster.ac.uk (P.S.M.D.); jwj.hamilton@ulster.ac.uk (J.W.J.H.); Sharma-P2@email.ulster.ac.uk (P.K.S.); a.vennard@email.ulster.ac.uk (A.S.M.V.); 2 Plataforma Solar de Almería—CIEMAT, PO Box 22, 04200 Tabernas, Almería, Spain; E-Mails: pfernandez@psa.es (P.F.-I.); mpolo@psa.es (I.P.-L.)

**Keywords:** heterogeneous photocatalysis, disinfection, water, healthcare associated infections, microorganisms, solar reactors

## Abstract

Photo-excitation of certain semiconductors can lead to the production of reactive oxygen species that can inactivate microorganisms. The mechanisms involved are reviewed, along with two important applications. The first is the use of photocatalysis to enhance the solar disinfection of water. It is estimated that 750 million people do not have accessed to an improved source for drinking and many more rely on sources that are not safe. If one can utilize photocatalysis to enhance the solar disinfection of water and provide an inexpensive, simple method of water disinfection, then it could help reduce the risk of waterborne disease. The second application is the use of photocatalytic coatings to combat healthcare associated infections. Two challenges are considered, *i.e*., the use of photocatalytic coatings to give “self-disinfecting” surfaces to reduce the risk of transmission of infection via environmental surfaces, and the use of photocatalytic coatings for the decontamination and disinfection of medical devices. In the final section, the development of novel photocatalytic materials for use in disinfection applications is reviewed, taking account of materials, developed for other photocatalytic applications, but which may be transferable for disinfection purposes.

## 1. Heterogeneous Photocatalysis

According to the IUPAC Gold Book, photocatalysis is the “*change in the rate of a chemical reaction or its initiation under the action of ultraviolet, visible or infrared radiation in the presence of a substance—the photocatalyst—that absorbs light and is involved in the chemical transformation of the reaction partners*.” A photocatalyst is defined as a “*catalyst able to produce, upon absorption of light, chemical transformations of the reaction partners. The excited state of the photocatalyst repeatedly interacts with the reaction partners forming reaction intermediates and regenerates itself after each cycle of such interactions*” [1]. In heterogeneous photocatalysis, the photocatalyst is present as a solid with the reactions taking place at the interface between phases, *i.e*., solid-liquid or solid-gas. For the purposes of this review we will be concerned with heterogeneous photocatalysis taking place at the surface of solid semiconductor materials, which act to absorb the photon energy and provide active sites for the adsorption of reactants. In semiconductor photocatalysis, the primary reactions are electrochemical oxidation or reduction reactions involving hole and electron transfer from the photo-excited semiconductor.

**Figure 1:**
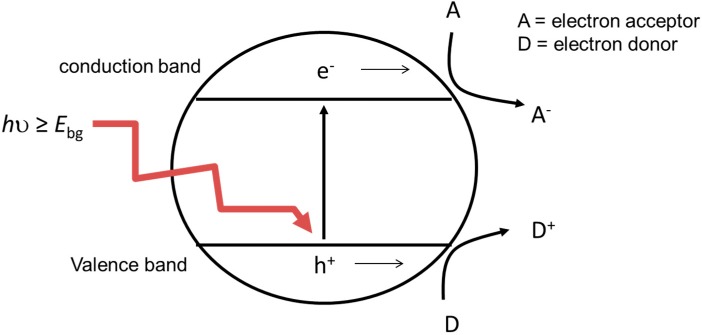
Schematic showing the basic mechanism of heterogeneous photocatalysis.

Figure 1 shows a schematic of the basic mechanism involved. The semiconductor is excited by the absorption of electromagnetic radiation with energy equal to or greater than the band gap energy (*E*_bg_). This results in the promotion of an electron from the valence band to the conduction band, leaving a positive hole in the valence band. The electron/hole pairs may recombine with the energy being re-emitted as heat or light, or the charge carriers can migrate to the particle surface. The conduction band electron can be passed on to an electron acceptor with a more positive electrochemical reduction potential than the conduction band edge potential. The valence band hole may accept electrons from donor species with a less positive electrochemical reduction potential than the valence band edge potential. Overall, these processes result in the reduction of an acceptor species and the oxidation of a donor species, where both reactions are driven by the potential difference generated by the absorption of electromagnetic radiation. The potential difference generated is close to the band gap energy of the semiconductor.

**Figure 2:**
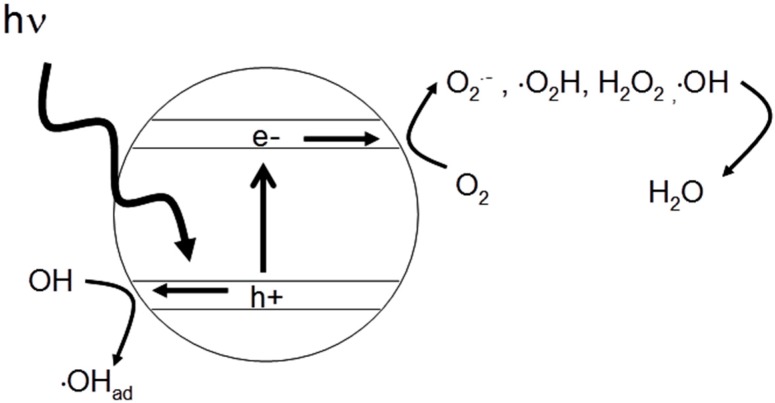
Schematic of photocatalytic mechanism on a titanium dioxide particle leading to the production of reactive oxygen species.

Reactions that are thermodynamically downhill (−ve ΔG) are photocatalytic, and reactions that are thermodynamically uphill (+ve ΔG) are photosynthetic. Nevertheless, it is common to use the term photocatalytic to describe up-hill reactions e.g., “photocatalytic” water splitting. For the purposes of photocatalytic disinfection, we are primarily concerned with surface redox reactions, which lead to the generation of reactive oxygen species (ROS). Figure 2 shows a general scheme for the production of reactive oxygen species where oxygen is acting as the electron acceptor and water or hydroxyl ions is acting as the electron donor. In this example, titanium dioxide (TiO_2_) is the semiconductor as the band edge potentials must be in suitable positions to drive the reactions of interest. Other semiconductor materials may be used—*vide infra*. The valence band hole may have an electrochemical reduction potential positive enough to oxidize water to yield hydroxyl radical and the conduction band should be negative enough to reduce molecular oxygen to yield superoxide radical anion, and via subsequent electron transfer, yield peroxide and hydroxyl radical. Overall, in the presence of oxygen and water, the photocatalytic mechanism can generate a mixture of ROS, which inactivate microorganisms or degrade organic chemical contaminants. For more detailed information on the physicochemical mechanisms of heterogeneous photocatalysis the reader should consult previously published reviews [2,3,4,5,6,7,8].

In this review, the mechanism of inactivation of microorganisms is discussed and two specific applications of photocatalytic disinfection are reviewed, *i.e*., the solar photocatalytic disinfection of water and photocatalytic coatings to combat healthcare associated infections. In the last section we review the development of novel photocatalytic materials with respect to the inactivation of microorganisms.

## 2. Mechanism of Photocatalytic Inactivation of Microorganisms

We are surrounded by microorganisms that colonize our skin, intestines and the environment we live in. In most cases we live in harmony with these microscopic entities, in some cases symbiotically, and we utilize them to produce food, degrade waste, generate essential chemicals and clean water. However, some microorganisms are the cause of disease and death. These pathogenic microorganisms cause diseases, such as Ebola hemorrhagic fever, typhoid, and cholera, and illnesses, such as flu and the common cold. They can be transmitted from person to person and sometimes from species to species, and transmission can be via direct contact, via body fluids, airborne in aerosol, infected surfaces, food, and water. Over the last century, humans have developed an armory of defenses against pathogenic microorganisms, including vaccines, antibiotics, disinfectants, food hygiene practices, water disinfection (chlorination, ozonation, UVC), sterilization, *etc.* Unfortunately pathogenic microorganisms rapidly evolve and adapt to resist our defense systems and we must utilize science and engineering to deal with existing and emerging pathogens. Heterogeneous photocatalysis has been shown to be effective for the inactivation of a wide range of pathogenic microorganisms, including some which are resistant to other methods of disinfection.

Since Matsunaga *et al*. first reported the inactivation of bacteria using TiO_2_ photocatalysis in 1985 [9] there have been more than 1000 research papers published in the area. The effectiveness of photocatalysis against microorganisms, including bacteria (cells [10,11], spores [12] and biofilms [11]), viruses [13], protozoa [14], fungi [15] and algae [16] has been investigated, and this work has been reviewed by McCullagh *et al*. [17], Malato *et al*. [8], and Robertson *et al*. [18]. In general photocatalytic disinfection in water requires minutes or tens of minutes of direct UVA exposure (using TiO_2_ as the photocatalyst) and it is considered to be quite a slow microbial inactivation process, as compared to e.g., UVC disinfection (seconds of direct exposure). The mechanism of photocatalytic inactivation is different from that of UVC disinfection. Whilst the majority of papers published in the area focus on the assessment of novel materials, new reactor systems or the effect of experimental parameters on the rate of inactivation, a significant number of studies have specifically investigated ROS interaction with the biological structures within microorganisms in an attempt to elucidate the mechanism resulting in the loss of organism viability. A review of the mechanisms involved in photocatalytic disinfection was conducted by Dalrymple *et al*. [19], however, the exact sequence of events leading to loss of viability is not completely understood. Continued insight into the mechanisms of attack of ROS on microorganisms will allow researchers to optimize materials and reactor design to improve the rate and efficacy of photocatalytic disinfection [20].

Upon excitation, a range of ROS can be generated at the semiconductor particle-solution interface. Of these the hydroxyl radical (HO^•^) has been suggested to be the primary species responsible for microorganism inactivation, however superoxide radical anion (O_2_^•−^), hydroperoxyl radical (HO_2_^•^) and hydrogen peroxide (H_2_O_2_) have been shown to contribute to the biocidal process [21]. Within biology the toxicity of “free radicals” is well known. Biological systems have enzymatic process to convert ROS into less toxic species (e.g., catalase and superoxide dismutase) and hydroxyl radicals play a pivotal role in the reaction of white blood cells with pathogens and apoptosis (programmed cell death). The prevalence of ROS within the environment in which microorganisms thrive and the evolution of defense systems against these active species are of particular relevance to photocatalysis. Unlike antibiotics, which target a specific biological process within the lifecycle of bacterial organisms (only), ROS attack is not specific to one site or an individual pathway. This permits the use of ROS generated by photocatalysis against a wide range of pathogens, and as important, the development of bacterial resistance to photocatalysis is considered to be almost impossible [22]. In photocatalytic disinfection, the ROS must be produced at concentrations greater than that which the microorganism can protect against in order to result in complete inactivation, thereby preventing bacterial recovery and re-growth.

ROS interaction with microbes typically occurs from the outside of the organism, in towards the sensitive metabolic processes and genetic materials within organisms. The “strength” of the outer layers of the organism effectively dictates the ability of the organism to survive, the thick protein, carbohydrate and lipid structures surrounding protozoa and bacterial spores present a more challenging target than viruses, fungi and bacteria—in that order [8]. Due to the relative ease of culture and detection, bacteria have been the most widely studied organisms, with *Escherichia coli* (*E. coli*) the primary species used.

Typically bacterial organisms are classified based upon the content of their outer cell layers, which surround an internal liquid based cytoplasmic matrix, comprising genetic material and the biochemical systems used for energy production, cell regulation and reproduction. The cytoplasm is bound by the cell membrane—a phospholipid bilayer, containing cross membrane proteins structures, which regulate transmission of chemicals into and out of the cytoplasm—and maintaining the integrity of this structure is of particular importance for bacterial viability. The cell membrane in Gram-positive bacteria is surrounded by a cell wall comprising a thick layer (20–80 nm) of porous peptidoglycan, responsible for structural integrity and the retention of Gram stain. Chains of lipoteichoic acid extend from the cell membrane through the cell wall and play roles in cell binding reactions. The cell wall in Gram-negative bacteria is much more complex. A thin layer of peptidoglycan (7–11 nm) is encapsulated by a second phospholipid bilayer, termed the outer membrane. This is populated with long extending chains of lipopolysaccharide, which elicit strong immune responses in animals, and as with the inner cell membrane contains cross membrane protein channels.

**Figure 3:**
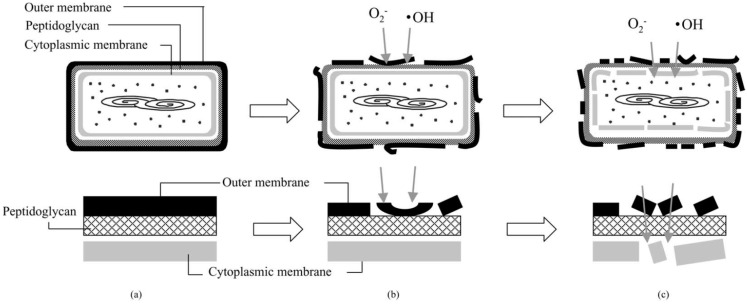
(**a**–**c**) Schematic illustration of the process of *E. coli* inactivation on photo-excited TiO_2_. In the lower row, the part of cell envelope is magnified. (Reproduced from Sunada, K.; Watanabe, T.; Hashimoto, K. Studies on photokilling of bacteria on TiO_2_ thin film. *J. Photoch. Photobio A* **2003**, *156*, 227–233 [27]).

Given the complexity of microorganisms, it is perhaps understandable why the full mechanism of photocatalytic inactivation is still unknown, however, the accepted sequence of events taking place during photocatalytic inactivation of microorganisms is that prolonged ROS attack results in damage of the cell wall, followed by compromise of the cytoplasmic membrane and direct attack of intracellular components (Figure 3). To this end, microscopy based studies have revealed the formation of pores within cell wall and cell membrane structures [23], the degradation of peptidoglycan has been reported [24], lipid peroxidation within phospholipids membranes demonstrated [24,25,26,27], degradation of porin proteins within cell membranes [28], the detection of intercellular compounds and genetic material exterior to the cell confirmed [9,29], and direct DNA damage reported via genetic analysis [30]. More specific modes of action discussed include membrane damage leading to inactivation of respiratory pathway chemistry [24] and loss of fluidity and increased ion permeability [27]. The general consensus relates to hydroxyl radical mediated lipid peroxidation of the outer cell wall components, and perhaps the most conclusive evidence for this was recently reported by Kubacka *et al.* [31]. Following a detailed and systematic investigation into the levels of a range of genetic and protein markers during of TiO_2_ photocatalysis the authors concluded that extensive radical induced cell wall modifications are the main factor responsible for the high biocidal performance of TiO_2_-based nanomaterials. Some workers have also proposed additional mechanistic action via “inside-out” processes, with Malato *et al*., describing that UVA irradiation of intracellular chromophores in the presence of oxygen could produce single stranded DNA breaks and nucleic acid modifications [8] and Gogniat and Dukan reporting DNA damage via Fenton reaction-generated hydroxyl radicals during bacterial recovery [32].

Whilst photocatalysis has great potential to be used as a biocidal technology, caution should to be exercised when conducting photocatalytic disinfection assays as organisms have the potential to recover from sub-lethal ROS exhibited stress and re-grow. Several authors have reported the need for complete inactivation of organisms and specific analysis to confirm that the treatment prevents subsequent bacterial re-growth [11,33]. The transfer of antibiotic resistance following sub-lethal photocatalytic treatment has recently been reported [34].

## 3. Solar Photocatalytic Disinfection of Water

Water is an important natural resource and safe drinking water is vital for human existence and good quality of life. Clean water resources are becoming depleted due to population growth, over-use of resources and climate change. Since the adoption of the Millennium Development Goals (MDG), the WHO/UNICEF Joint Monitoring Programme for Water Supply and Sanitation has reported on progress towards achieving Target 7c: “reducing by half the proportion of people without sustainable access to safe drinking water and basic sanitation” [35]. Although the world met the MDG drinking water target, 748 million people, mostly the poor and marginalized, still lack access to an improved drinking water source. Almost a quarter of these (173 million) rely on untreated surface water, and over 90% live in rural areas. It is estimated that there will be 547 million people without an improved drinking water supply in 2015. Many more are forced to rely on sources that are microbiologically unsafe, leading to a higher risk of contracting waterborne diseases, including typhoid, hepatitis A and E,polio and cholera [35,36,37,38]. The WHO estimated that in 2008 diarrheal disease claimed the lives of 2.5 million people [39]. For children under five, this burden is greater than the combined burden of HIV/AIDS and malaria [40]. A total of 58 countries from all continents reported a cumulative total of 589,854 cholera cases in 2011, representing an increase of 85% from 2010. The greatest proportion of cases was reported in Latin America and Africa [41].

Although the MDG drinking-water target refers to sustainable access to safe drinking water, the MDG indicator—“use of an improved drinking water source”—does not include a measurement of either drinking water safety or sustainable access. This means that accurate estimates of the proportion of the global population with sustainable access to safe drinking water are likely to be significantly lower than estimates of those using improved drinking water sources.

Piped-in water supplies are a long-term goal and interventions to improve water supplies at the source (point of distribution) have long been recognized as effective in preventing waterborne disease. Recent reviews have shown household-based (point-of-use) interventions to be significantly more effective than those at the source for the reduction of diarrheal diseases in developing regions (possibly due to contamination of water between collection and use). As a result, there is increasing interest in such household-based interventions that can deliver the health gains of safe drinking water at lower cost. Household water treatment and safe storage (HWTS) is one option for improving the quality of water for consumption within the home, especially where water handling and storage is necessary and recontamination is a real risk between the point of collection and point of use. Living conditions in many humanitarian crises also call for effective HWTS. The practice of household water treatment and safe storage can help improve water quality at the point of consumption, especially were drinking water sources are distant, unreliable or unsafe. HWTS is a stop-gap measure only and does not replace the obligation of a service provider to supply access to safe drinking water. Household water treatment (HWT) methods include boiling, filtration, adding chlorine or bleach, and solar disinfection.

In 2008, Clasen and Haller reported on the cost and cost effectiveness of household based interventions to prevent diarrhea [42]. They compared: chlorination using sodium hypochlorite following the “Safe Water System” (SWS) developed and promoted by the US Centers for Disease Control and Prevention (CDC); gravity filtration using either commercial “candle” style gravity filters or locally fabricated pot-style filters developed by Potters for Peace; solar disinfection following the “SODIS” method in which clear 2 L PET bottles are filled with raw water and then exposed to sunlight for 6–48 h; and flocculation disinfection using Procter and Gamble PUR^®^ sachets, which combine an iron-based flocculent with a chlorine-based disinfectant to treat water in 10 L batches. They concluded that household-based chlorination was the most cost-effective. Solar disinfection was only slightly less cost-effective, owing to its almost identical cost but lower overall effectiveness. Given that household-based chlorination requires the distribution of sodium hypochlorite, solar disinfection has a major advantage in terms of non-reliance on chemical distribution.

Sunlight is freely available on Earth and the combined effects of heat and UV from the sun can inactivate pathogenic organisms present in water. Of course, there are a number of parameters that affect the efficacy of the solar disinfection (SODIS) process, including the solar intensity and temperature (weather conditions), and the level and nature of the contamination (some pathogens are more resistant to SODIS than others). One approach to SODIS enhancement is the use of heterogeneous photocatalysis.

Furthermore, the presence of some water pathogens is also an issue in developed regions. Urban wastewater treatment plants (WWTP) are among the main sources of antibiotics’ release into the aquatic environment worldwide, and therefore these are considered as hotspots of antibiotics and promote the genetic selection and generation of antibiotic resistant bacteria in water [43]. Therefore, the occurrence of antimicrobial resistant pathogens in the effluents of urban WWTP is evidence that conventional secondary water treatment cannot effectively control them. A number of bacterial species have been identified which exhibit acquired resistance to one or more types of antibiotics [44]. It is also recognized that chlorination of drinking water as tertiary treatment affects the fraction of antibiotic-resistant bacteria by potentially assisting in microbial selection, and regrowth or reactivation of antibiotic-resistant bacteria after chlorination in wastewater [45]. Nowadays, one of the major scientific challenges is to reduce the incidence of waterborne disease globally and also address the increasingly virulent and antibiotic resistant pathogens such as vanomycin-resistant enterococci (VRE) and methicillin-resistant *Staphylococcus aureus* [46]. Therefore alternative new technologies for water disinfection are needed to mitigate these problems at all levels.

The photocatalytic enhancement of solar water disinfection has been successfully proven in many occasions using solar light, either natural or artificial, to photo-excite TiO_2_ nanoparticles suspended or immobilized against a wide number of model water pathogens, which go from bacteria (*E. coli*, *E. faecalis*, *total coliforms*, *Salmonella*, *Pseudomonas*, *etc.*), virus (MS2 phage, RNA bacteriophage, phi164, *etc.*), spores of bacteria as *Bacillus subtillis* and fungi like *Fusarium*, *Candida albicans*, *Aspergilllus niger*, *Phytophthora*, *etc.*, and parasites like *Cryptosporidium parvum* [10,12,14,47,48,49,50].

The configuration of the catalyst in the reactor can significantly alter in the disinfection results. Typically there are two ways to use the TiO_2_ for water treatment purposes, (i) as aqueous suspensions of TiO_2_ particles; and (ii) as immobilized TiO_2_ over an inert and robust support which should be resistant to the photocatalytic process and also to the hydrodynamic conditions (flow and pressure) in the photo-reactor during the process. The configuration of the catalyst in the photo-reactor depends, among others factors, on the final application of this water treatment. If the system is designed for drinking water purification for human consumption, the use of suspended TiO_2_ particles, as a part of a routine intervention for improving the potability of water at the house-hold level (point-of-use water treatment) may not be acceptable due to concerns over toxicity of nanomaterials [51] and the photocatalyst particles would have to be removed before consumption; However, one should point out that TiO_2_ is used as a food additive (E171) and is found in a wide range of food products, including coffee creamer, and the use of a few mg L^−1^ for the disinfection of water would result in a lower intake of TiO_2_ than the average US citizen. To avoid any complication of using dispersed titania, one can utilize the photocatalyst as an immobilized system, but this will result in lower rates of disinfection or more complex systems with higher cost [8,52].

**Figure 4:**
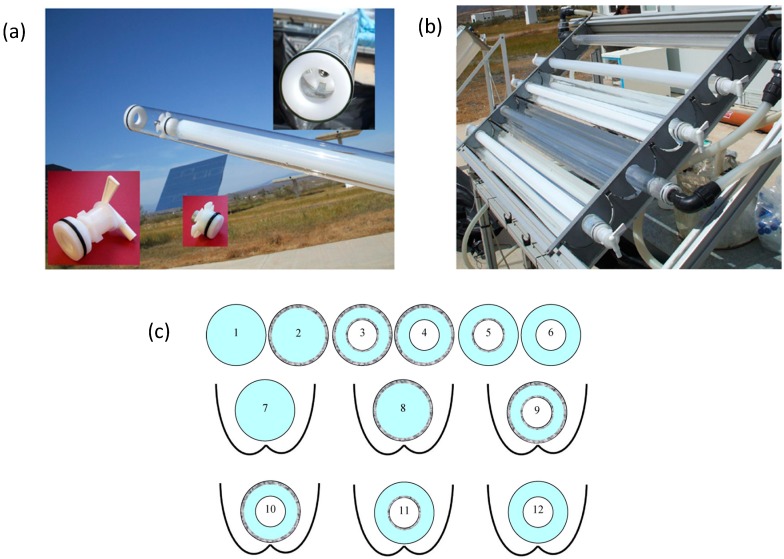
Photographs showing the double tube configuration with internal tube cap and the valve for external tube (**a**) and the solar photocatalytic reactor with and without CPC during disinfection tests (**b**). Schematic cross-section representation of the different reactor configurations tested in the solar reactor (**c**), (1)/(7) uncoated single tube without/with CPC; (2)/(8) coated single tube without/with CPC; (3)/(9) coated double tube without/with CPC; (4)/(10) coated external–uncoated internal without/with CPC; (5)/(11) coated internal–uncoated external without/with CPC; (6)/(12) uncoated double tube without/with CPC (reproduced from Alrousan, D.M.A.; Polo-López, M.I.; Dunlop, P.S.M.; Fernández-Ibáñez, P.; Byrne, J.A. Solar photocatalytic disinfection of water with immobilized titanium dioxide in re-circulating flow CPC reactors. *Appl. Catal. B* **2012**, *128*, 126–134 [54]).

Different types of supported TiO_2_ for improvement of the efficiency of solar water disinfection like cylinders, pills, balls, mesh, *etc*., have been investigated, for example TiO_2_ deposited on glass rashing rings inside a tubular reactor of a CPC solar prototype was compared with slurry systems [53]. The maximum efficiency was found with the suspended TiO_2_ particles reactor, due to the better surface contact between bacteria and catalyst. However, fixed-bed reactors lead to inactivation rate quite close to that of the slurry. It is not only the high titania surface area of this configuration which is responsible for the bacteria inactivation but the important contribution of the mechanical and osmotic stress have to be considered. The main advantage of the fixed-bed TiO_2_ catalyst is the non-requirement for recovery of the catalyst following treatment; however the longevity of the catalyst must be considered. In the study by Sordo *et al*. [53], the catalyst was tested for ten reaction cycles without deactivation being observed. Most of these immobilized systems can be re-used for several cycles, as long as the catalyst is not fouled by inorganic species such as phosphates and sulfates. More work is needed on photocatalyst longevity under operational conditions. Alrousan *et al*., tested solar photocatalytic (SPC-DIS) and solar disinfection (SODIS) of water at pilot scale using different reactor configurations with and without immobilized TiO_2_ (Evonik Aeroxide P25) (Figure 4). The model organism used was *E. coli*. The use of compound parabolic collectors improved the SODIS and SPC-DIS of water; however, the improvement was less significant compared to the improvements reported previously for SODIS in static batch reactors. Kinetic fitting yields a log-linear component (first order rate constant). The following order was found for *k* where coated refers to TiO_2_ coating and the equals sign indicates no significant difference; uncoated external = coated internal ≥ double coated tube ≥ uncoated double tube. It is known that *E. coli* is inactivated by SODIS and it may be a “soft” target for comparing the effectiveness of SODIS *vs*. SPC-DIS. Nevertheless, photocatalysis presents advantages in terms of the non-recovery of inactivated organisms and the inactivation of SODIS resistance organisms [54].

### 3.1. Photocatalytic Materials Tested under Solar or Solar Simulated Conditions

Most research studies on photocatalytic disinfection have been carried out with the commercial TiO_2_ *Evonik Aeroxide P25* (formerly *Degussa P25*) photocatalyst. Nevertheless, some research has been carried out with pure anatase and doped titanium dioxide. For solar applications, visible light active materials are desirable, to increase the photon absorption beyond the solar UVA spectrum, which is only *ca.* 4% of the global solar component reaching the Earth surface. However, the smaller band gap, while absorbing a greater number of solar photons, gives a narrower voltage window to drive the redox reactions at the interface. Furthermore, metal sulfide semiconductors, which absorb in the visible region of the spectrum, tend to undergo photo-anodic corrosion [52]. Unfortunately, the number of publications concerning the photocatalytic activity of these materials for the inactivation of microorganisms is limited. The UV activity of undoped TiO_2_ may be greater than the visible light activity of a doped material. Therefore, for solar applications, the efficiency should be tested under simulated solar irradiation or under real sun conditions. Rengifo-Herrera and Pulgarin reported on the photocatalytic activity of N, S co-doped and N-doped commercial anatase (Tayca TKP 102) TiO_2_ powders towards phenol oxidation and *E. coli* inactivation [55]. However, these novel materials did not present any enhancement as compared to *Evonik Aeroxide P25* under simulated solar irradiation. They suggest that while the N or N, S co-doped TiO_2_ may show a visible light response, the localized states responsible for the visible light absorption do not play an important role in the photocatalytic activity. More research is required to determine if visible-light active materials can deliver an increase in the efficiency of photocatalysis under solar irradiation. Nowadays, considering cost, chemical and photochemical stability, availability, and lack of toxicity, the most suitable catalyst reported to date for the disinfection of water is TiO_2_.

**Figure 5:**
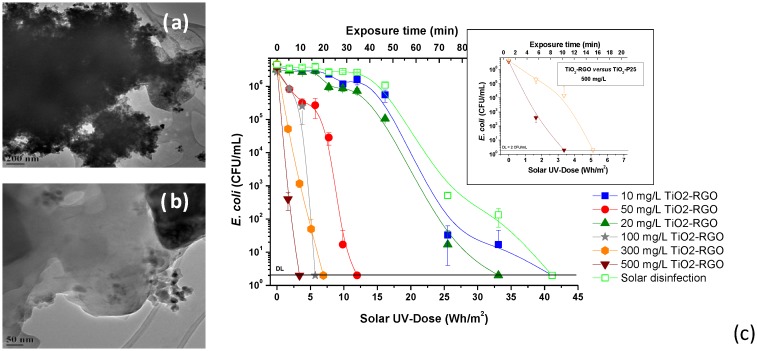
(**a**) TiO_2_-GO aggregate before photoreduction; (**b**) TiO_2_-RGO after UV assisted photoreduction and (**c**) *E. coli* inactivation at several TiO_2_-RGO concentrations. Figure inserts shows efficiency of TiO_2_-RGO and TiO_2_-P25 on the *E. coli* inactivation (reproduced from Fernández-Ibáñez, P.; Polo-López, M.I.; Malato, S.; Wadhwa, S.; Hamilton, J.W.J.; Dunlop, P.S.M.; D’Sa, R.; Magee, E.; O’Shea, K.; Dionysiou, D.D.; Byrne, J.A. Solar Photocatalytic Disinfection of Water using Titanium Dioxide Graphene Composites. *Chem. Eng. J*. **2015**, *261*, 36–44 [20]).

Some researchers have been investigating the use of titanium dioxide—reduced graphene oxide (TiO_2_-RGO) composites to improve the photocatalytic efficiency. This material may be easily synthesized by the photocatalytic reduction of exfoliated graphene oxide (GO) by TiO_2_ (*Evonik Aeroxide P25*) under UV irradiation in the presence of methanol as a hole acceptor. TiO_2_-RGO composites were compared to suspended TiO_2_ (P25) for the disinfection of water contaminated with *E. coli* cells and *F. solani* spores under natural sunlight (Figure 5). Rapid water disinfection was observed with both *E. coli* and *F. solani*. An enhanced rate in the *E. coli* inactivation efficiency was observed with the TiO_2_-RGO composite compared to P25 alone. In this work, the materials were evaluated with filtered sunlight; the major part of the solar UVA was cut-off (λ > 380 nm). In this case, a much greater time was required for inactivation of *E. coli* with TiO_2_ P25 but the same inactivation rate was observed for the TiO_2_-RGO indicating visible light activity, which could be attributed to singlet oxygen production by TiO_2_-RGO composites, which would lead to *E. coli* inactivation [20].

Other researchers have investigated the efficacy of several nanomaterials under natural sunlight for water disinfection. The materials were employed as suspended nanoparticles in stirred batch reactors using mainly anatase phase (P25 and PC500), or mainly rutile phase (Ruana), and samples of Bi_2_WO_6_; these two last materials having the advantage of absorbing more solar photons. The photocatalyst activities were compared with solar disinfection (without catalyst) under natural sunlight using *E. coli* as model microorganism. This work proved that the adding of any kind of photo-catalyst to the water accelerated the bactericidal action of solar irradiation and led to complete disinfection during treatment times that varied from 60 to 150 min. The photocatalytic disinfection efficiency was not enhanced by the increase of catalyst concentration above 0.5 g/L for P-25, PC500 and Bi_2_WO_6_, where 10^6^ CFU/mL were completely inactivated within 5 min, 30 min and more than 150 min of solar exposure under clear sky, respectively. An increase of the concentration to 1 g/L slightly decreased the total inactivation time. Rutile (Ruana) catalyst behaves differently where the optimal concentration was lower than for the other titania materials and agglomeration of the particles occurred as the concentration of catalyst was increased. Anions and cations released during *E. coli* inactivation was monitored *i.e.*, sodium, ammonium, potassium, magnesium, calcium, chlorine, sulfate and nitrate, as indicators of damage to bacterial cells. Only ammonium and potassium were formed with varied quantities depending on the photocatalyst used. Ammonium can be formed by the photocatalytic oxidation of amino acids, which composed the protein present in cells membranes. The appearance of K^+^ during bacterial inactivation is in agreement with other studies that demonstrated that the loss of K^+^ was followed by the loss of cell viability, while other contributions suggested that damage in cell membrane occurs after or with the K^+^ loss and this can also cause cell death [56].

The morphology of photocatalyst nanoparticles has been also investigated, as well as the relationship between well-tuned TiO_2_ photocatalyst and their exposed facet with the photocatalytic activity against *Fusarium* spores in water. Four TiO_2_ morphologies were tested: nanotubes (NT), nanoplates (NPL), nanorods (NR) and nanospheres (NS). The solar photocatalytic properties were compared with the disinfection of spores of *Fusarium solani* in water. The solar inactivation of the resistant spores of *Fusarium* in water was demonstrated to be related to the exposed TiO_2_ facets. At very low concentrations of photocatalyst, the inactivation of *F. solani* over TiO_2_ nanospheres showed the best disinfection efficiency with respect to the others morphologies. The simultaneous presence of formic acid during photocatalytic disinfection and decontamination showed that the presence of this organic acid strongly retards the disinfection reaction in the case of TiO_2_ nanospheres while formic acid degradation occurred simultaneously with *F. solani* inactivation in the case of TiO_2_ NT [57].

Other new materials have been developed and investigated for improvement of the solar photocatalytic efficiency. As an example, Ag-BiVO_4_ composites were synthesized and their photocatalytic disinfection activity was tested against *E. coli* under visible light (λ > 420 nm) [58]. This work reported that the deposition of silver nanoparticles on the surface of BiVO_4_ led to a significant improvement of the photocatalytic activity. These composites had a red shift edge on the UV-Vis absorption, which increased with the amount of deposited silver. Photocatalytic inactivation of *E. coli* in the presence of Ag/BiVO_4_ resulted in the total disinfection of the cells (10^7^ to less than 1 CFU/mL within 3 h). This photocatalytic activity was stable in repeated runs under natural sunlight. They attributed this significant photocatalytic enhancement of Ag/BiVO_4_ to the effect of metallic silver nanoparticles. Ag particles can act as an electron sink on the surface of semiconductors (BiVO_4_), which prevents the recombination of e^−^/h^+^ pairs. When the metal and semiconductor are in contact, a Schottky barrier is formed. The Schottky barrier height is equal to the energy barrier that electrons need to overpass to migrate from the metal to the semiconductor. Electrons will naturally migrate from the semiconductor to the metal towards reaching the equilibrium chemical potential. This results in the accumulation of electrons on the metal (negative charges) and excessive positive charges on the semiconductor interface, therefore in an efficient separation of e^−^/h^+^ pairs during the photocatalytic process. This may explain that Ag nanoparticles on BiVO_4_ form a Schottky barrier at the interface and enhance the photoactivity of the semiconductor composite, which promotes the separation of photo-induced e^−^/h^+^ pairs for the generation of reactive oxygen species. The development of novel photocatalytic materials will be discussed in more detail later in Section 5.

### 3.2. Solar Reactor Design and Applications

There are several approaches to improving the efficacy of solar disinfection and solar photocatalysis disinfection of water. These enhancements should consider the following aspects:

(i) Increasing the appropriate solar photons entering the photo-reactor system.
(ii) Improving the efficacy of the treatment against certain resistant water pathogens.
(iii) Increasing the total volume of treated water for a certain treatment time (solar exposure).
(iv) Reducing complexity of the water treatment system and decreasing the user dependency of the process.
(v) Using low-cost systems based on simple designs and local and cheap materials to construct the water disinfection systems for application in developing countries.
(vi) Design optimized photo-reactor systems that avoid post-treatment recovery or re-growth of microorganisms and prevent recontamination of treated water due to inappropriate handling or storage.

The first research on reactors using compound parabolic collectors (CPC) with TiO_2_ solar photocatalysis for water disinfection was reported by Vidal *et al.* [59]. This solar photo-reactor had a 4.5 m^2^ CPC solar collector that maximized the collected solar radiation for long exposure periods (several hours). They showed a 5-log decrease of *E. coli* and *Enterococcus faecalis* in 30 min of solar treatment with suspended TiO_2_ (P25; 0.5 g/L) and natural solar irradiation (average UVA irradiance: 25 W/m^2^). They also reported an economic analysis of this technology for future application not only to solar photocatalytic disinfection, but also to decontamination of organic pollutants. Mcloughlin *et al.* [60] evaluated three geometrical configurations of solar mirrors at lab-scale, solar photo-reactors with aluminum reflectors consisting of compound parabolic, parabolic and V-groove profiles and showed that they enhanced the bactericidal effect of the natural solar radiation using *E. coli* as the model microorganism. The CPC was found to be more efficient than the parabolic or V-groove profiles. They also demonstrated that bacterial photo-inactivation using sunlight alone can be enhanced by low loadings of TiO_2_ (P25 at few mg/L) suspended in the water.

**Figure 6:**
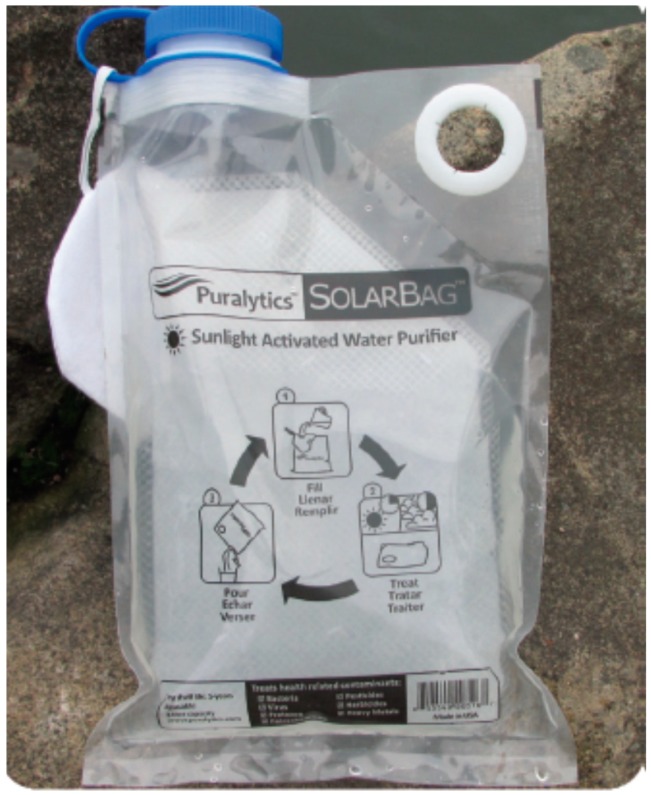
Solar bag commercially available from Puralytics, which utilizes photocatalysis (from Puralytics website [61]).

Up to now, there are few commercial systems based on solar disinfection or solar photocatalytic disinfection. One example of a commercial system is the Solar Bag available from Puralytics (see Figure 6) [61]. Prototypes and bench scale reactors are continuously being developed by different groups with the aim of developing effective, reliable and low-cost solar reactors for water purification. These contributions are investigating the main factors affecting the disinfection process and performance under real conditions of solar radiation, real contaminated water (drinking water, wastewater, *etc.*), naturally occurring microorganisms, and in the presence of naturally organic and inorganic matter. One of the major challenges in this field is to create a system that disinfect and decontaminate water at the same time. A large part of the research on solar reactors for water disinfection has been carried out using the mere action of solar radiation, *i.e*., the synergistic effect of solar thermal heating and the detrimental effects of solar UVA (and some small percentage of UVB). Other research is mainly focused in the photocatalytic acceleration of the inactivation of microorganisms. It is important to put together both fields, the concepts of both processes to better accomplish the task of efficient solar reactor systems for water disinfection and decontamination. One of the most critical criteria for good solar photo-reactor performance is the increase of the volume of treated water output in a reasonable solar treatment time. To address this objective one must take into account the following limiting technical aspects.

**Figure 7:**
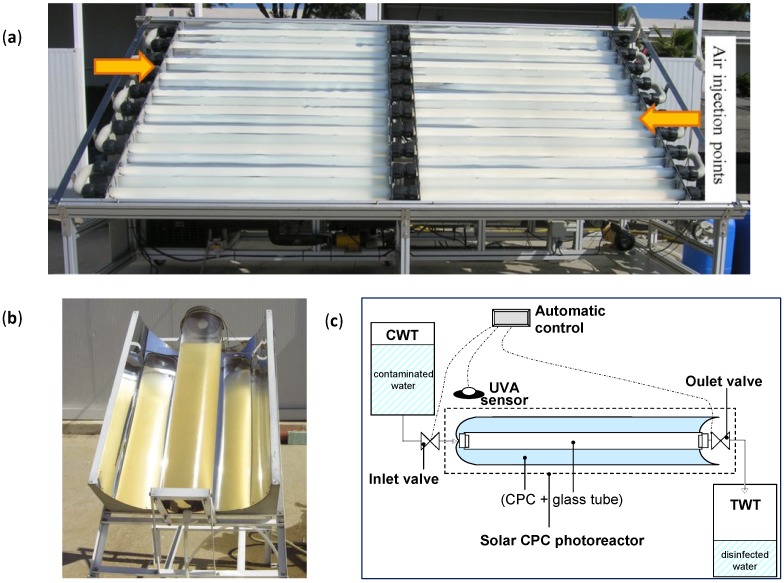
(**a**) Images of front view of the solar 60 L-CPC reactor at PSA facilities (4.5 m^2^ of collector mirrors) with air injection points indicated; (**b**) Enhanced SODIS batch reactor filled with 100 NTU turbid water; (**c**) Schematic of the sequential batch system.

(i) The incoming photon-flux inside solar photo-reactor must be as high as possible (using either CPC solar mirrors or other low-cost reflectors, which increase the solar light collection). It should be efficiently and homogeneously distributed in the photo-reactor water, even for large volumes of water. Therefore light scattering and absorption of water samples is a key factor, thus there must be a compromise between the optical properties of the water and the physical path length of the photo-reactor (diameter if it is a tube, or depth if it is a pond or raceway). If the UV-Vis transmission of the water is good enough (turbidity < 10 NTU), the optical reactor path length (*i.e*., diameter of the tubular photo-reactor) can be increased until 10–20 cm (Figure 7b) [62]. The use of solar mirrors accelerates the disinfection rate, the increase in solar irradiance decreases the treatment time and accelerates the disinfection rate, but not necessarily in a linear proportionality manner [63].
(ii) When the water does not have good UV-Vis transmission (*i.e*., when suspended TiO_2_ is used or turbid waters are treated), then the reactor tube must be reduced to few centimeters and the large volume requirement may be accomplished by connection of several photo-reactor modules [64].
(iii) The use of immobilized photocatalyst is desirable, mainly for drinking water applications. In this case, the water flow rate must be low enough to increase the residence time required to achieve the desired disinfection in a single pass (the contact time will depend on the nature of the pathogen and the immobilized photocatalyst used) so that the recovery mechanisms of some microorganisms cannot effectively work after the treatment within the residence time. This important limitation has been widely investigated [53,65,66] with the conclusion that the flow rate must be low enough (only a few L/min) to ensure that a minimum lethal dose of energy is delivered before the water circulates to dark regions of the reactor. In the dark regions of the reactor, bacteria may recover and re-grow when the damage produced by the disinfecting agent (either by direct action of solar photons or by hydroxyl radicals) is not strong enough to achieve total inactivation (*i.e*., complete disinfection) and desired disinfection efficiencies may not be achieved [63,65,66]. Therefore, the design of the reactor must be done with prior knowledge of the lethal solar energy dose required so that complete disinfection is achieved in one run. Obviously, this energy requirement for complete killing depends strongly on several parameters like water composition, turbidity and nature of the biological contamination. A number of examples of the influence of the presence of ions, organic matter and natural occurring bacteria present in wastewater are available in the literature [65,67,68].
(iv) There are limitations of solar disinfection (without any photocatalyst) when it is scaled-up through the use of large batch volumes or continuous flow recirculation reactors [63]. Increasing the flow rate has a negative effect on inactivation of bacteria, as at a given time point there needs to be maximum exposure of bacteria to UV to ensure inactivation as compared to having bacteria exposed to sub-lethal doses over a long period of time. When the water is kept static under solar light it is constantly illuminated and hence the required uninterrupted UV dose can be easily achieved. With continuous flow systems, the lethal dose can be delivered but in an intermittent manner and complete inactivation is not observed. This statement has important implications for those attempting to scale-up solar systems through the use of pumped, re-circulatory, continuous flow reactors. If the operational parameters are set such that the microbial pathogens are repeatedly exposed to sub-lethal doses of solar radiation followed by a period within which the cells have an opportunity to recover or repair, complete inactivation may not be achieved.
(v) In photocatalytic disinfection, the electron acceptor is normally dissolved oxygen, which is easily available from the air. In the case of static batch systems, the concentration of dissolved oxygen will be rapidly depleted and must be replenished to maintain photocatalytic activity. Furthermore, the solubility of oxygen in water is reduced by temperature. This must be taken into consideration as the temperature within solar irradiated reactors can reach 55 °C. New designs of reactor must address the need for replenishment of dissolved oxygen in photocatalytic disinfection systems. This was investigated in the recent contribution of Garcia-Fernandez *et al*. [67]. These authors investigated the influence of temperature and dissolved oxygen using a solar 60 L-CPC reactor with suspended TiO_2_ (0.1 g/L) (Figure 7a). They injected air in the reactor pipeline (160 L/h) and evaluated several controlled (fixed) temperatures (15, 25, 35 and 45 °C) for the photocatalytic inactivation of two models of water pathogen, *E. coli* (vegetative cell model of fecal contamination) and *Fusarium solani* (a spore model of resistant fungi). In this work, they also assessed the effect of the chemical composition of the water and compared real urban wastewater with synthetic ones. They observed that increasing the water temperature, from 15 to 45 °C, had clear benefits on the disinfection rate for both pathogens. They also observed that air injection led to an important enhancement on the inactivation efficiency, which was even stronger for *F. solani* spores, the most resistant pathogen evaluated. The composition of the water matrix significantly affected negatively the efficiency of the photocatalytic treatment, showing a better inactivation rate in simulated urban wastewater effluent than for real urban wastewater effluent.
(vi) An alternative to enhance photocatalytic performance may be to introduce other oxidants e.g., H_2_O_2_, persulfate radical, *etc.* however, this would give rise to a dependence on consumable chemicals which may be affordable or undesirable depending on the final application of the system.
(vii) Other aspects like reducing the user dependence of the process or making the system as cheap and robust as possible are worthy to consider so that reactors can be used worldwide for solving a number of issues related with water safety, mainly for human consumption purposes in developing countries. The automated SODIS reactor developed by Polo-Lopez *et al.* [66] addressed some practical problems associated with increasing the treated water output using a continuous flow concept. This novel sequential batch photo-reactor was designed and constructed with the aim of decreasing the treatment time, increasing the total volume of water treated per day and reducing user-dependency. The photoreactor incorporated a CPC concentration factor of 1.89 and the treatment time was automatically controlled by an electronic UVA sensor (Figure 7c). The feedback sensor system controlled the gravity-filling of the reactor from an untreated water reservoir, and controlled the discharge of the treated water into a clean reservoir tank following receipt of the pre-defined UVA dose. The reactor was tested using *E. coli* in well water under real sun conditions in Southern Spain. They found that this system would permit processing of six sequential batches of 2.5 L each day (*i.e*., 15 L of solar purified water each per day). The system is modular, therefore it may be scaled up to allow several CPC photoreactors to be used under the control of a single UVA sensor. For example, six systems like this could produce around 90 L of potable water per day (for several households), and it could produce approximately 31,500 L during a typical year. Of course static systems do not present a good option when using immobilized photocatalyst, as mass transfer limitations will predominate unless a mechanism for forced convection and mixing is introduced.

## 4. Photocatalytic Coatings for Healthcare Applications

There is a need for new and/or complimentary methods of disinfection to combat the on-going problem of health care associated infections (HCAI). A healthcare associated infection is defined as an infection contracted during the hospital stay [69]. The estimated cost of HCAI to the National Health Service (NHS) in the UK is at least £1 billion per year. HCAI’s cause added stress and inconvenience to patients, and it has been estimated that around 5000 deaths per year can be attributed to HCAI’s [70]. Recent reports suggest that the incidence of certain strains has decreased but infection from other strains is increasing [71]. Healthcare patients are more susceptible to infection as they may be already ill with low immunity, their own endogenous flora can present as opportunistic pathogens, and they are at risk from infection from other pathogens in the healthcare environment which may be transmitted via the air, water or from contact with contaminated persons or surfaces [11]. Although the extent of this risk has not been clearly established, it is known that hospital surfaces and/or medical devices can become contaminated with infectious pathogens, which can potentially play a role in the spread of HCAI. This is further substantiated by reviews, which recommend increased cleaning to reduce incidence of HCAI [72,73].

One of the key factors in HCAI transmission is the ability of some pathogens to remain viable from days to months on surfaces and medical devices, therefore acting as a perpetual source of infection [74] and becoming a reservoir of infection [75]. Routine decontamination and cleaning is essential in the healthcare environments to reduce the risk of transmission of infection, and deep cleaning is essential following an outbreak of infection [76]. It has been shown that shared medical equipment can become contaminated with bio-burden (a population of viable infectious agents contaminating a medical device [77] and this increases the risk of transmission of infection to patients [70]. For example, stethoscopes (which are commonly used) can be a vector if not decontaminated properly [78]. An additional source of HCAI’s is transmission from other medical implements, such as contaminated surgical equipment, or invasive medical devices, such as catheters [79,80]. There are a number of factors which determine the role surfaces play in the spread of infectious agents: the longevity of the organisms; the frequency of which the site is touched; and the concentration of pathogens on the surface, *i.e*., if it is high enough to result in spreading to patients [72]. The most common hospital pathogens are Methicillin-resistant *Staphylococcus aureus* (MRSA), *Clostridium difficile*, *Escherichia coli* (*E. coli*) (and its resistant form extended beta lactamase or ESBL *E. coli*) and Vancomycin-resistant *enterococci* (VRE). Near patient sites should be cleaned and disinfected. These include bed rails, equipment stands and environmental surfaces, *etc.*, common hand touch sites, such as door handles, and all medical appliances. Photocatalytic coatings may provide an additional means of surface disinfection and decontamination to help reduce the incidence of HCAI’s.

Photocatalytic TiO_2_ coatings and composites have been utilized for commercial application in tiles, paving slabs and self-cleaning glass [81,82]; however, there are few commercial medical applications, although self-cleaning tiles by TOTO are reported to be effective for use in hospitals, yet published data and case studies are not readily found [83]. There are a range of approaches to the formation of TiO_2_ coatings on surfaces including sol-gel, chemical vapor deposition (CVD) and physical vapor deposition (PVD). Anatase is usually reported to be the most active crystal phase of titania and formation normally requires elevated temperatures between 225 °C and 550 °C. Therefore coating temperature labile materials with photocatalytically active titania is a challenge.

Some researchers have investigated photocatalytic coatings for disinfection and decontamination of devices and materials including catheters [79], lancets [84], dental adhesives [85] and implants [86]. Other researchers have investigated the use of photocatalytic coatings for environmental surface disinfection [87,88].

### 4.1. Self-Disinfecting Coatings for Environmental Surfaces

In 2003, Kühn *et al*. [87] reported on the use of UV activated photocatalytic coatings for surface disinfection. As UV activation was required for the TiO_2_ coatings, and levels are low under ambient conditions, they used UV sources with light guides and a light-guiding sheet to ensure UV irradiation of the surface. They investigated the inactivation of *E. coli*, *Pseudomonas aeruginosa*, *Staphylococcus aureus* (*S. aureus*), *Enterococcus faecium* and *Candida albicans*. The photocatalytic coating was a thin film of TiO_2_ deposited on Plexiglass and the surface was irradiated from above and below. Their results indicated a 6-log_10_ reduction in colony forming units (CFU) after 60 min for *P. aeruginosa*, *S. aureus* and *E. faecium*. For *C. albicans* a 2-log_10_ reduction in CFU over 60 min was observed. *E. coli* and *P. aeruginosa* showed the best responses in this experiment as they had the greatest reduction efficiencies out of all the bacteria. The controls without a photocatalyst (which were subject to UVA light only) showed a bacterial reduction after 60 min for *E. coli* and 10 min for *P. aeruginosa*. Analysis by light microscopy indicated that photocatalytic bacterial inactivation results from direct damage to the cell walls. For surface disinfection, a 3–5-log_10_ reduction in the amount of infectious agents is normally adequate. It is not clear how the UV irradiation could be delivered in a real healthcare environment without risk to staff and patients.

Page *et al*., carried out research on the antibacterial activity of titania and silver-titania composite films prepared using a sol-gel dip coating method on glass slides [88]. Clinically relevant strains of bacteria were used as the model organisms, including *S. aureus*, *E. coli* and *B. cereus.* The titania and silver-doped titania nanoparticulate thin films were created using a dip-coating procedure followed by heat treatment at 500 °C. The photocatalytic activity of the films was determined using stearic acid degradation monitored by FTIR. The samples and their controls were initially illuminated using a 254 nm germicidal UV lamp for 30 min to allow time for activation and disinfection of the films. The disinfection experiments were undertaken with irradiation from black-light tubes with main emission at λ = 365 nm (1.4 mW/cm^2^). Irradiation times ranged from 2 to 6 h. The films showed photocatalytic activity for the inactivation of all strains studied, with the Ag-TiO_2_ films showing better inactivation rates as compared to the TiO_2_.

The photocatalytic disinfection properties of sputtered TiO_2_ films were tested by Miron *et al*. [89]. They tested the bactericidal ability of their films against *Diplococcus pneumoniae*, *Staphylococcus aureus* and *E. coli*. The bacteria were grown and then transferred to the films. After 1 h UV light irradiation (light intensity 1 mW/cm^2^) initial bacterial membrane destruction was observed on the sputtered TiO_2_ films. After 6 h irradiation the bacteria were completely destroyed; however, 6 h irradiation is a significantly long time for UV photocatalytic disinfection. When compared to the uncoated glass substrate, there was no noticeable destruction of bacteria after 6 h.

Dunlop *et al*. [11] investigated the inactivation of clinically relevant pathogens on photocatalytic coatings. A method was developed to assess the disinfection efficiency of photocatalytic surfaces which allowed the determination of pathogen viability as a function of treatment time, the assessment of the surface for viable surface bound organisms following disinfection and measurement of the re-growth potential of inactivated organisms. The developed method was used to investigate the inactivation of extended-spectrum beta-lactamase (ESBL) *Escherichia coli*, methicillin resistant *Staphylococcus aureus* (MRSA), *Pseudomonas aeruginosa* and *Clostridium difficile* spores using immobilized films of titania nanoparticles (*Evonik Aeroxide P25*). A 99.9% reduction in viability (3-log kill) was observed for all bacterial cells within 80 min of photocatalytic treatment (under UVA irradiation). Complete surface inactivation was demonstrated and bacterial re-growth following photocatalytic treatment was not observed. More than 99% inactivation (2.6-log reduction) was observed when the photocatalytic surfaces were contaminated with *C. difficile* spores.

As TiO_2_ photocatalysis is only activated by UV photons, this reduces its potential for use in the disinfecting of environmental surfaces under solar or ambient lighting conditions. Therefore, attempts have been made to create visible light active (VLA) photocatalysts by, for example, doping TiO_2_ with nonmetals. Wong *et al*. tested the efficiency of carbon doped (C-TiO_2_) and nitrogen doped TiO_2_ (N- TiO_2_) created by ion-assisted electron beam evaporation [90]. It was found that the N-TiO_2_ had better bactericidal ability than the C-TiO_2_. Using the N-TiO_2_ for the bactericidal experiments inactivation of human pathogens *Shigella flexneri*, *Listeria monocytogenes*, *Vibrio parahaemolyticus*, *Staphylococcus aureus*, *Streptococcus pyogenes*, and *Acinetobacter baumannii* as well as laboratory strain *E. coli* were tested. They achieved less than a 1-log_10_ reduction (90%) in the *E. coli* population using an incandescent light and N-TiO_2_, however this was after only 25 min illumination (light intensity 3 × 10^4^ lux) therefore this is a relatively good inactivation time for VLA disinfection. Prior to this, it was found that when testing a range of visible light intensities, this higher value (3 × 10^4^ lux) showed the best inactivation. Inactivation of the human pathogens was less successful as they were only reduced by 50% and the authors conclude they were possibly more resistant to N-TiO_2_ mediated treatment due to presence of enzyme systems.

Mitoraj *et al*., tested visible light activated photocatalysts for use on surfaces in areas that require clean and sterile conditions e.g., a hospital [91]. A variety of pathogens (*Escherichia coli*, *Staphylococcus* *aureus*, *Enterococcus faecalis*, *Candida albicans*, *Aspergillus niger*) were tested in suspension using C-TiO_2_ and TiO_2_ modified with platinum (IV) chloride, irradiated with a high-pressure mercury lamp (of varying light intensities 1.8 W/cm^2^ and 1.0 W/cm^2^). The inactivation of the pathogens follows the same order as listed above. According to the author, this is because the density and complexity of the cell wall increases from the gram-negative *E. coli*, to gram positive *S. aureus* and *E. faecalis* to the *C. albicans* and *A. niger* fungi. The authors also tested the antimicrobial activity of immobilized catalysts, which is more relevant to a self-disinfecting surface. The bactericidal efficiency of an immobilized catalyst is influenced by several factors including the ease with which light and oxygen can access the photocatalyst surface, the distance between pathogens and surface and the ability of the radicals to penetrate the microbial cells. When the C-TiO_2_ was immobilized, it was not capable of inactivating either *E. coli* or *S. aureus*. The TiO_2_-platinum (IV) chloride suspension was immobilized onto plastic plates and showed fast inactivation of *E. coli*, when irradiated with a halogen lamp of light intensity 0.2 W/cm^2^. After 30 min, 98% of the bacteria were inactivated. In the dark the TiO_2_-platinum (IV) chloride was also capable of inactivating the bacteria meaning it has some bactericidal ability of its own, however this was at a slower rate. The reason for the bactericidal ability of the catalyst under irradiation is thought to be due to platinum species becoming desorbed from the catalyst surface and subsequently becoming partially reduced to platinum (II) in the presence reducing agents (such as photogenerated e^−^). Both platinum (II) and platinum (IV) complexes can inhibit bacterial DNA, RNA and protein synthesis. Therefore it was proposed that the fast activity of the photocatalyst under light irradiation was mainly due to the toxicity of the catalyst when irradiated, however the authors do not state if the disinfection process was further enhanced by the photogeneration of ROS (^•^OH, *etc.*). The ability of the catalyst to inactivate bacteria in the dark shows it also has inherent toxic qualities. The other microorganisms were not tested using this method; however the results show that this immobilized photocatalyst has potential to become a self-disinfecting surface, however the disinfection affect was probably not due to VLA photocatalysis. It is somewhat surprising that the authors did not test the efficiency of TiO_2_ loaded with Pt nano-clusters, as the latter has been reported to show high photocatalytic efficiency in many studies.

Dunnill *et al*. tested the efficiency of S-TiO_2_ and N-TiO_2_ for the inactivation of *E. coli* under white light illumination. The films were prepared using atmospheric pressure chemical vapor deposition (APCVD) [92]. The light source used was one common in UK hospitals, a fluorescent lamp which had a luminosity of 5965 lux when measured at 20 cm from the lamp. The TiO_2_ thin films were prepared using APCVD using a custom CVD reactor, with a nitrogen or sulfur source being added for the doping. The sulfur or nitrogen precursor was added during the vapor stage to the deposition chamber. The authors found that good interstitial doping of nitrogen was achieved when low quantities were added, therefore giving good photocatalytic activity. Nitrogen incorporation at 0.13 at.% gave the best photocatalytic activity and sulfur incorporation of 0.1 at.% gave the best activity. The absorption edge for the *N*- and *S*-doped samples were found to be 2.9 eV and 3.0 eV, respectively, while the undoped TiO_2_ had an optical band gap of 3.2 eV (normal for anatase). The samples were tested for their photocatalytic disinfection properties using *E. coli*. The best efficiency was found on samples pre-irradiated for 24 h as this provides a clean surface before application of bacteria. After bacterial inoculation onto the samples, they were further illuminated with white light for 24 h and the nitrogen-doped sample appeared to perform slightly better than the sulfur-doped sample with kill rates of 99.9% and 99.5% respectively. The undoped TiO_2_ and glass only substrates showed little or no kill, showing that the visible light was not able to activate the TiO_2_. Although this study reports VLA, it is a very slow process. The indoor lighting with doped catalysts was able to inactivate the bacteria, with the N-doped sample slightly outperforming the *S*-doped sample; however the time taken for disinfection was very long (24 h). Nevertheless, if photocatalytic coatings can provide a residual disinfection mechanism, this may reduce the risk of transmission of HAI via contaminated surfaces.

More recent work by Dunnill *et al*., investigated the ability of nanoparticulate Ag loaded titania thin films to kill bacteria under hospital lighting conditions [93]. It is well known that Ag has some antimicrobial activity and nano-Ag is already used in existing products such as catheters, ventilator tubing and surfaces. Silver ions are toxic to microbes and since they are able to move from the antimicrobial material into the microorganisms the cells become damaged by the ions. The purpose of the coatings made in their study was to determine if they were visible light active and to exploit the antibacterial effects of both the silver and the photocatalysis using a common hospital light source. The films were fabricated using a sol–gel method to coat glass slides with TiO_2_ in the form of anatase. Ag nanoparticles were then loaded onto the surface by UV photo-reduction of Ag onto the TiO_2_ from AgNO_3_ solution to give Ag-TiO_2_ substrates. The substrates were then annealed at 500 °C. Optical absorbance gave an optical band gap of 2.8 eV. Experiments were carried out to determine photo-induced superhydrophilicity, degradation of stearic acid and the antibacterial effect of the films. *E. coli* and epidemic MRSA (EMRSA-16) were used as the model microorganisms. With the MRSA a significant enhancement in the inactivation was observed with the Ag-TiO_2_ film under white light irradiation as compared to the TiO_2_ film. For *E. coli*, a 99.996% (4.4 log_10_ CFU) decrease in the number of viable bacteria was observed on the Ag-TiO_2_ films incubated under white light for 6 h, compared with the TiO_2_ films incubated under the same light conditions for the same time period; However, a similar decrease in the bacterial number was demonstrated with the Ag-TiO_2_ film in the absence of light indicating that the observed inactivation of *E. coli* was due to the Ag in the dark.

An important consideration is that the rate of photocatalytic disinfection is rather slow and the presence of organic contaminants will compete for ROS. Therefore, photocatalytic coatings for the disinfection of environmental surfaces under ambient lighting conditions should be additional to normal cleaning routines and may provide some residual surface disinfection to reduce the risk of transmission of infection.

### 4.2. Photocatalytic Coatings for the Disinfection and Decontamination of Medical Devices

Catheters and other medical tubes are extensively used in hospitals to administer medicine or nutrients into arteries and drain fluids or urine from the urethra or digestive organs. Indwelling catheters have the potential to cause infection and around 20% of healthcare acquired infection occurs to the urinary tract, potentially by the use of catheters. Catheters are also an attractive reservoir for microorganisms. To combat this some catheters with antibacterial action have been developed, for example those with silver impregnated in them. However, this is not a long-term solution as silver ions are released from the wall of the material and will eventually wear out. Ohko *et al*. developed TiO_2_ coated silicone catheters in an effort to exploit the antibacterial and self-cleaning properties of the coating when exposed to UV light [79]. Silicone is one of the most common materials for catheter manufacture; therefore this type of catheter was used. However, because this material has low wettability, it is difficult to coat the TiO_2_ directly onto it. Therefore the catheter was first dipped in sulfuric acid to improve the overall wettability then dipped into a sol containing titanium dioxide. The existence of the coating on the catheter still allowed it to remain adequately flexible and hard for practical use. The authors found that the coating on the inside of the catheter could be irradiated by low-intensity UV light when transmitted from the outside. Therefore it is expected that the photocatalytic disinfecting qualities could be experienced on the inside as well as the outside of the catheter. The degradation of methylene blue was tested and it was found that on the inside of the tube the methylene blue was bleached, meaning that the UV light was transmitted to the inside of the tube and photocatalysis took place. The bactericidal efficiency was also tested using *E. coli* and it was found after 60 min irradiation the survival ratio had decreased to a negligible amount. The authors state this kind of catheter has potential for use in intermittent catheterization. Dunlop *et al*. studied the efficacy of photocatalytic disinfection to inactivate *Staphyloccocus epidermidis* cells within a biofilm [11]. Following 3 h of UVA irradiation, 96.5% of the biofilm cells on the TiO_2_ film were determined to be non-viable. Importantly, inactivation of cells throughout the 3–4 μm thick biofilm was observed.

Lancets, used to prick the finger for self-monitoring of blood glucose levels, and other needles must be completely sterile as they enter the body. The use of needles and in particular lancets has increased in recent years due to the rise of diabetes. Two important factors in the development of needles are to create a low piercing resistance and also the need to sterilize the needles by safe and low cost methods. There are some problems with current sterilization methods of lancets, for example the need for specialized equipment for gamma-ray sterilization. Therefore Nakamura *et al*., assessed whether TiO_2_ photocatalysis could be a low cost alternative sterilization method and studied the benefits of adding a TiO_2_ coating to the lancets [84]. In this study the medical grade lancets were coated with a uniform nano-layer of TiO_2_ by sputter deposition. The antimicrobial activity was assessed using *E. coli* K12. After 45 min of UV irradiation 83% of the bacteria were inactivated. The uncoated control lancet showed 33% inactivation and lancets in the dark showed no inactivation. Un-annealed lancets showed similar results to the uncoated control lancet when subject to UV light, meaning that the un-annealed TiO_2_ coating was not photoactive and that any killing was probably due to the UV light. The lancets must have a low lancing resistance this to reduce the pain induced when someone pricks their finger. This is important, as diabetics must carry out this task several times a day. The annealed TiO_2_ film showed low lancing resistance.

Oka *et al*. studied the ability of a titanium dioxide coating on percutaneous implants to inhibit bacterial colonization [86]. Metal pins are widely used for the application of skeletal traction or for external fixation devices in the repair of orthopedic fractures. These pins cannot be completely sterile as they are not isolated from the environment but rather are a link from outside of the skin, which is usually colonized with bacteria, to the bone, which is not normally colonized [94]. A considerable number of fracture fixations, either external of percutaneous, can become infected. Pin tract infection is a considerable complication and is normally combated with antibiotics. However, due to the increasing resistance of certain pathogens, like MRSA, antibiotics may not prove effective for inactivating bacteria. If bacteria is not inhibited and infection around the pin occurs this can cause pin loosening, pin removal or chronic osteomyelitis. If the fixation is changed from external to internal there will be a risk of deep infection, because the pin will penetrate the skin barrier. Another problem of implant-related infection is the risk of biofilm formation. Biofilm is a complex build-up of many layers of bacteria, which surround themselves in a protective exopolymeric fluid. Biofilms have an advantage over normal free-living bacteria in that they are extremely resistant to treatment from antibiotics [75]. To avoid the formation of biofilm and pin tract infection in general, bacteria must not be allowed to adhere and colonize on the implant. The authors therefore created a TiO_2_ photocatalyst, which was processed from a pure titanium plate via direct oxidation. MRSA was used as the test pathogen, as it is one frequently associated with percutaneous tract infection. After 60 min UVA irradiation of the photocatalyst the survival rate was negligible. The authors also acknowledge that there were several limitations to this study including the way they applied a large inoculation of bacteria directly to the implant is not how bacterial colonization occurs if the infection occurred clinically. Also, UVA irradiation cannot reach the subcutaneous part of the pin. An animal model was also used to test colonization of bacteria onto the implant and it was found that fewer bacteria colonized the photocatalyst surface than the pure titanium surface. Hydrophilicity is another important factor of TiO_2_ photocatalysis. Oxygen vacancies are created when the photogenerated holes react with superoxide anion and oxygen atoms are ejected. The oxygen vacancies then become occupied with water molecules, which make the surface hydrophilic. It is thought a hydrophilic surface deters bacteria from adhering to it therefore the hydrophilicity combined with the self-cleaning effect of the TiO_2_ photocatalyst, which would inactivate and remove bacteria attached, would provide an effective and functional coating. Tsuang *et al*. also studied the use of TiO_2_ photocatalytic coatings on percutaneous implants [94]. The coating was applied to stainless steel plate by a sol-gel dip coating method. The authors found a significant reduction in the amount of *E. coli* colonies above the TiO_2_ coated metal plate. Other pathogens were tested in suspension, however they would not provide useful results whenever the TiO_2_ coating would be immobilized onto a metal pin.

Shiraishi *et al*. researched the bactericidal ability of TiO_2_ coated metal implants against *S. aureus*, which is associated with many surgical site infections (SSI) [95]. SSI are normally a result of contamination during surgery. Consequently, there is a need to develop methods to reduce bacterial infection linked to implants. Pure titanium and medical grade stainless steel (SU316) sample disks were synthesized with TiO_2_ using a plasma source ion implantation (PSII) system. The authors state that this method is very feasible for creating TiO_2_ coated implants as it can deposit the films completely and uniformly over three dimensional surfaces and there is little other existing evidence of the bacterial ability of PSII deposited-TiO_2_ metal implants. The photocatalytic activity of the TiO_2_ coated stainless steel and titanium as well as the control was assessed via the degradation of methylene blue. It was found there was no degradation on the control disk irradiated with UVA (peak wavelength 352 nm/light intensity 2.0 mW/cm^2^) or the dark control TiO_2_ coated disks that were not subject to UVA. Degradation was only observed on disks with UVA irradiated TiO_2_. The light control (UVA irradiation with no photocatalyst) showed a gradual decline in the viability of the bacteria. The most appreciable results were seen on the TiO_2_ coated discs under UVA irradiation. TiO_2_ films on titanium showed a clear reduction in cell viability and complete kill after 90 min irradiation. TiO_2_ on stainless steel showed a quicker reduction in cell viability than that of the titanium supporting TiO_2_ and complete kill was achieved after 60 min. The authors conclude that TiO_2_ deposited by PSI could have the potential to reduce the occurrence of SSIs with regards to medical implants and the photocatalytic bactericidal ability to inactive *S. aureus* has many potential benefits for sterilizing contaminated surfaces of bio-implants.

Medical polymers are also at risk from contamination. Therefore improving the antimicrobial properties of polymers is important. Polymethyl methacrylate (PMMA) is commonly used in the manufacture of medical implants such as in the fabrication of ophthalmic intraocular lenses (IOL) as well as dentures and bone cement. Because it is optically transparent it can be used for replacement intraocular lenses in the eye when the original lens is removed in the treatment of cataracts [96] and also for tonometer tips, which are used to measure intraocular eye pressure [97]. This material is useful because of its good mechanical properties, mouldability and use for ophthalmic rehabilitation. The existing properties of PMMA could further be enhanced by the addition of antibacterial TiO_2_ coating that had high transmittance in the visible region and prove useful for ophthalmic applications. Polymers have proven difficult to coat with TiO_2_ however, as evaporated inorganic coatings do not adhere successfully to polymers. Another obstacle is the need for polymer surfaces to be kept at a relatively low temperature, as they are thermally sensitive. Su *et al*., created translucent TiO_2_ films onto PMMA using a sol-gel dip coating method [98]. Prior to this the PMMA surfaces were activated by pre-treating with low pressure DC glow discharge plasma. After coating, the PMMA was dried in an oven for 30 min at 60 °C. XRD analysis found that anatase TiO_2_ was present. The bactericidal ability properties of the TiO_2_ coated PMMA was tested using *E. coli* BL21 and *S. aureus* with what the authors describe as “indoor natural light” (average intensity at 365 nm of 143.9 μW/cm^2^ and at 297 nm of 6.7 μW/cm^2^ during daytime antibacterial test hours). After 15 min of illumination, 50% of the *E. coli* BL21 had been inactivated. After 2 h, none of the bacteria were detected on the agar plate. The controls: PMMA in the dark, PMMA with light and TiO_2_/PMMA in the dark did not show any reduction in the number of bacteria. The authors also found that after bacterial adhesion tests, the amount of adherent *S. aureus* was decreased by 89%–92% and adherent *E. coli* was decreased by 96%–98%. The PMMA alone was not capable of decreasing the amount of adherent bacteria meaning that the induced surface hydrophilicity of the TiO_2_ may have given it better anti-adhesion properties. The authors conclude that their research created successful translucent TiO_2_ coatings onto PMMA that had efficient antibacterial and anti-adhesion properties. The translucence may limit the applications of these types of coatings.

Suketa *et al*. were one of the first groups to report on the use of photocatalytic coatings for dental implants [99]. Oral implants normally have better clinical success when the surface of the implant is altered to improve the integration between bone and implant. One of these alterations is to increase the surface roughness; however an increased surface roughness is thought to increase the risk of a bacterial infection, which can lead to a build-up of plaque. The presence of plaque on a surface for a long period of time can result in peri-implantitis which affects both soft and hard tissues around the osseointegrated implants resulting in the development of implant pockets and loss of supporting bone. Therefore to compromise between the need for a rough surface and fact that rough surfaces potentially harbor more bacteria than smooth ones, an alternative sterilizing method is needed to clean the rough surface and/or remove the formation of plaque biofilm. The authors suggest that TiO_2_ photocatalyst coating would be advantageous for two reasons, the photocatalyst normally is more efficient with a high surface area and an implant with a roughened surface will have a large surface area. *Actinobacillus actinomycetemcomitans* and *Fusobacterium nucleatum* were the test pathogens, which are thought to play a key role in the causation of peri-implantitis. The photocatalyst coatings were created using PSII. It was found that the surface roughness of the anatase and control aluminum oxide were not significantly different. The bacterial tests showed that the TiO_2_ disk subject to UVA illumination inactivated 100% of the bacteria after 120 min. There was also a reduction in colony forming units with light control sample; however it was much less than that of the photocatalytic test. A TiO_2_ surface should not prevent the osseointegration of implant and bone, and it has been shown that TiO_2_ surfaces created by PSII did not prevent osseointegration in a rabbit. The photocatalytic surface prepared in this study was shown to have good surface roughness therefore it should be able to attach successfully to bone and not prevent osseointegration. After implantation of the implant, flap elevation would be needed to sterilize the contaminated surface because the UVA radiation would not be able penetrate thick human tissues. The authors highlight some challenges that need to be addressed including; 120 min of UVA is too long for clinical use, and that more intense UV would damage human tissue. Furthermore, bacterial tests would be needed on the other pathogens associated with peri-implantitis. Nonetheless their research has shown the potential for TiO_2_ photocatalysis in this kind of application.

Another type of dental application investigated was the use of TiO_2_ in dental adhesives for restoration procedures and other purposes [85]. Many dental restorations are unsuccessful and a replacement is needed due to the onset of dental caries. The adhesives may provide a site that allows bacteria to attach onto and colonize; therefore the development of materials that have bactericidal ability at the tooth-composite boundary may be beneficial in combating dental caries. Hence there is a potential for the use of TiO_2_ coatings. TiO_2_ can increase bioactivity, in this case the interfacial bonding of a material to bone tissue by means of formation of biologically active hydroxyapatite (HA) [85]. Bioactivity is important as it can improve the bond between tooth and material. Many dental adhesives are not bioactive therefore adding a photocatalytically active TiO_2_ may also prove useful as a bioactive layer. The dental adhesives in this study were fabricated to include TiO_2_ nanoparticles and the bactericidal ability and bioactivity was studied. The commercially available dental adhesive material was mixed with the P25 nanoparticles containing different weights. *Staphylococcus epidermidis*, a gram-positive species, was used as the test pathogen as it is normally part of the skin flora, forms biofilms and is also resistant to antibiotics. The different light sources were used: a low intensity UV light of 1.2 mW/cm^2^ and a strong UV light, used with a filter to block wavelengths below 320 nm, to produce a UVA intensity of 7.5 mW/cm^2^. The low intensity light was used for 30 or 120 min whereas the high intensity light was used for 7 or 10 min. The nanoparticle adhesives (NP adhesives) containing 10% and 20% P25 subject to the low intensity irradiation for 120 min both showed significant reduction in the amount of colony forming units (CFU) after 18 h incubation. After 30 min low intensity irradiation and 12 h of incubation there was no regrowth of bacteria. However for the same conditions and 18 h incubation the bacterial regrowth was extensive showing that the bacteria must have experienced non-fatal damage during the 30 min, which only delayed or slowed the regrowth. The control glass plate subject to low intensity light showed no bactericidal effect. Using high intensity UV light, efficient bacterial elimination was observed on 20% NP adhesive after 10 min irradiation. Due to the negative impact of UV radiation on tissues within the body and in particular the gums, the intensity should be kept as low as possible and also localized to just the tooth area possibly by using a light guiding tube. It is likely that the adhesive would be sterilized once during or after the curing process by administering the UV light. As the adhesive would then be covered by a composite it would be difficult for the light to reach the photocatalytic coating therefore reducing the antimicrobial effect. For a clinically feasible irradiation time, which is less than 60 s [85], a high intensity would be needed. This could be achieved by using a regular dental curing light and therefore result in a superior antibacterial effect. The coatings also proved to be bioactive, after a week in simulated body fluid the NP adhesives showed build-up of HA crystals, which is typical of bioactive behavior.

Creutzfeldt-Jakob disease (CJD) is a rare and fatal condition that affects the brain. CJD appears to be caused by an abnormal infectious protein called a prion. These prions accumulate at high levels in the brain and cause irreversible damage to nerve cells. While the abnormal prions are technically infectious, they are very different to viruses and bacteria. Prions are not destroyed by the extremes of heat and radiation used to kill bacteria and viruses, and antibiotics or antiviral medicines have no effect on them [100]. Sporadic CJD is the most common type of CJD although it is still very rare, affecting only one to two in every million people each year in the UK. There were 104 recorded deaths from sporadic CJD in the UK during 2013. Variant CJD is likely to be caused by consuming meat from a cow that has been infected with a similar prion disease called bovine spongiform encephalopathy (BSE, “mad cow disease”). There have been 177 recorded cases of variant CJD in the UK to date and there was one recorded death from the condition in the UK during 2013. There were eight deaths from familial CJD and similar inherited prion diseases in the UK during 2013. Iatrogenic CJD can occur if instruments used during brain surgery on a person with CJD are not properly cleaned between each surgical procedure before re-use on another person. There was one death from iatrogenic CJD in the UK during 2013 [101]. Paspaltsis *et al*., reported on the inactivation of prion protein using titanium dioxide photocatalysis in 2006 [102]. They used P25 in suspension under UVA irradiation to treat solutions containing recombinant prion proteins PrP (normal isoform) and PrP^SC^ (scrapie, abnormal isoform), including suspensions of inoculated brain homogenate. The analysis of the protein was undertaken using electrophoresis and immunoblotting. Experiments were also carried out to determine infectivity using an animal model. TiO_2_ photocatalysis under UVA irradiation and the addition of H_2_O_2_ showed complete degradation of PrP^SC^ after 12 h, as determined by Western Blots. The *in vivo* assessment of infectivity of inoculated brain homogenates indicated that the infectivity titre was substantially reduced following photocatalytic treatment.

Ahmed, Byrne and Keyes reported on the degradation of β-amyloid peptides on the surface of Ag-TiO_2_ films [103]. The degradation of β-sheet peptides is relevant as the abnormal conformation of the prion protein is mainly β-sheet. TiO_2_ films were prepared on stainless steel using rf magnetron sputtering and the films were then modified by the photocatalytic reduction of Ag^+^ from solution. The adsorption of β-amyloid (1–42), photolytic and photocatalytic degradation of the peptide was studied using Raman spectroscopy, X-ray photoelectron spectroscopy (XPS), and atomic force microscopy (AFM). The Ag-TiO_2_ films were mainly anatase and the Ag was predominantly Ag (>0%). Ag loading of the TiO_2_ markedly enhanced the Raman signal (*ca.* 15-fold), but caused significant changes to the protein spectrum indicating non-specific binding of β-amyloid side chain residues to the silver. The amide modes remained well-resolved and were used to estimate the conformational change induced in the protein by the silver. Raman analysis showed an increase in the intensity of the band at ~1665 cm^−1^ assigned to the disordered conformation of the β-amyloid, suggesting that the adsorption at the silver sites induced conformational changes in the peptide. Contaminated surfaces were exposed to UVB irradiation and further conformational changes in the β-amyloid were observed which mildly inhibited amyloid fibril formation.

Table 1 summarizes some of the papers reviewed, the photocatalyst used, the model microorganism used, and the proposed application.

## 5. Novel Photocatalytic Materials

Although the bulk of photocatalytic disinfection concerns TiO_2_ as the photocatalyst, other materials have been investigated [104]. ZnO is the second most commonly investigated photocatalytic material after TiO_2_ and a number of ZnO based compounds have been reported for photocatalytic disinfection. For instance, ZnO nanoparticles [105] and nanorod films [106] have been investigated for the inactivation of *E. coli* disinfection under UV irradiation (ZnO is also a wide band semiconductor). The main goal of novel material development has been to produce visible light active materials. Two slightly different strategies have been employed; the development of narrower band gap materials and modification/sensitizing of existing wider band gap materials. Principally materials with visible light activity should benefit from increased efficiency under solar irradiation due to the greater number of photons and improved activity under indoor or ambient lighting conditions.

**Table 1:** Showing application, type of photocatalyst, microorganism tested and advantages/disadvantages.

Proposed Application	Photocatalyst	Microorganisms Tested	Reference
Environmental surfaces and medical devices	Evonik Aeroxide P25 immobilized on glass slides	extended-spectrum beta-lactamase(ESBL) *Escherichia coli*, methicillin resistant *Staphylococcus aureus* (MRSA), *Pseudomonas aeruginosa* and *Clostridium difficile* spores	[11]
General environmental cleaning	TiO_2_ (P25) on glass	*E. coli*, *P. aeruginosa*, *S. aureus*, *E. faecium*, *C.albicans*	[87]
Coating for hard surfaces in hospital environment	TiO_2_ and Ag-TiO_2_ on glass slides via sol-gel dip coating method	*E. coli*, *S. aureus*, *B. cereus*	[88]
Self-disinfecting catheters	TiO_2_ dip coated onto silicone catheters	*E. coli*	[79]
Lancet	TiO_2_ layer created by sputter depositions	*E. coli*	[84]
Percutaneous implant	Direct oxidation of pure Titanium plates	MRSA	[86]
Metal pin for external/percutaneous fixation	TiO_2_ coated on stainless steel via sol-gel dip coating	*E. coli*	[93]
Metal implant	Stainless steel and titanium coated with TiO_2_ via PSII	*S. aureus*	[94]
Intraocular lenses	PMMA plasma pre-treated and dip-coated with TiO_2_	*E. coli* *S. aureus*	[97]
Dental implant	Titanium disc coated via PSII	A*. actinomyce-temcomitans* *F. nucleatum*	[98]
Dental adhesives	TiO_2_ nanoparticles mixed with commercially available dental adhesives	*S. epidermidis*	[85]
Antibacterial surface	TiO_2_ films sputter deposited onto silicon wafers	*Diplococcus pneumoniae*, *S. aureus*, *E. coli*	[89]
VLA coatings for environmental surfaces	N-doped TiO_2_ created by ion-assisted electron beam evaporation	*S. flexneri*, *L. monocytogenes*, *V. para-haemolyticus*, *S. aureus*, *S. pyogenes*, A. *baumannii*, *E. coli*	[90]
Self-disinfecting hospital surfaces	C-TiO_2_ and TiO_2_ modified with platinum (IV) chloride	*E. coli*, *S. aureus*, *E. faecalis*, *C. albicans*, *A. nige*r	[91]
Antimicrobial healthcare surfaces	Sulfur and nitrogen doped titanium dioxide composites create via APCVD	*E. coli*	[92]
Antimicrobial healthcare surfaces	Ag loaded TiO_2_ films created via sol-gel	*E. coli*, EMRSA	[93]

### 5.1. Narrow Band Gap Materials for Visible Light Activity

The majority of new visible light active photocatalysis research is focused on water splitting/hydrogen evolution reactions. However the reaction path from water to hydrogen is complex involving a number of ROS intermediates, making many of these materials capable of disinfection through ROS attack and other mechanisms. Therefore, the literature concerned with water splitting literature is rich with possible new photocatalysts for disinfection applications.

**Figure 8:**
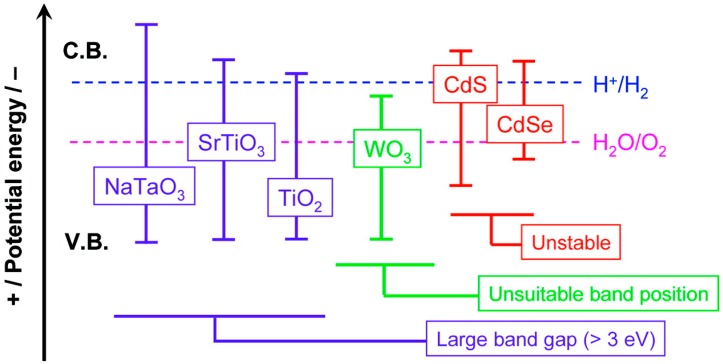
Band gap energies and band edge potentials for different photocatalytic materials with respect to the water splitting couples (with permission from Maeda, K.; Domen, K. New Non-Oxide Photocatalysts Designed for Overall Water Splitting under Visible Light. *J. Phys. Chem. C* **2007**, *111*, 7851–7861 [113]).

The path to development of visible light active photocatalyst materials started with testing of simple binary materials consisting of two elements such as WO_3_ Fe_2_O_3_, *etc.* These materials can produce reactive oxygen species under visible irradiation [107] and have been reported to elicit disinfection [108,109]. However, many of the narrow band gap photocorrode under irradiation [110,111]. Narrow band gap (1.8–2.5 eV) materials include Fe_2_O_3_, SiC CdS, and Cu_2_O. Wider band gap ( 2.6–2.8 eV) materials such as WO_3_ and MoO_6_ are more stable the small change in band gap for these materials, compared to TiO_2_ (3.0–3.2 eV), results in limited improvements in efficiency for visible light activity [112]. Figure 8 shows the band gap and band edge potentials for different semiconductor materials, which have been investigated for water splitting.

Metal carbide, nitride and sulfide materials generally have narrower band gaps than oxide materials but they tend to photocorrode in aqueous media. Some non-metal oxide materials have been investigated e.g., ZnIn_2_S_4_ was reported to show visible light photocatalytic activity for the inactivation of *E. coli* inactivation (under electrochemical bias) [114].

The search for stable visible light active materials photocatalyst materials have been pursued from both a design inspired and combinatorial chemistry approaches. Using combinational chemistry, a large number of potential materials with varying ratios of elements can be rapidly generated and, although assessment of their photocatalytic activity may require a lot of effort [115]. Design-based material developments concentrating on specific photocatalyst properties have been useful in guiding strategies for further material development.

Some visible light active photocatalyst materials have been designed using transition metal elements known to absorb strongly in the visible region due to d-d transitions. Iron centered catalysts prepared using combinatorial routes have been particularly popular [115]. New visible light active materials including CuFeO_2_ [116], CuY_x_Fe_2-x_O_4_ [117], LaFeO_3_ [118] have been reported as photocatalysts. Strontium titanate ferrite has been reported to have antibacterial activity under visible light irradiation [119]. Other transition metal ion centered photocatalyst materials reported include: manganese centered CuMn_2_O_4_, and ZnMn_2_O_4_ [120], Chromium centered BaCr_2_O_4_ [121] and SrCrO_4_ [122], Cobalt centered NiCo_2_O_4_ [123], CuCo_2_O_4_ [117], LaCoO_3_ and La0.9Sr0.1CoO_3_ the latter of which demonstrated photocatalytic disinfection against *E. coli* [124].

Novel photocatalysts based on vanadium centers are an interesting group of photocatalyst materials as vanadium oxides generally have narrow band gaps < 1 eV. However, when nanostructured to isolate a body centered cubic (BCC) phase this material shows an optical band-gap ~2.7 eV, resulting in photocatalyst, with high quantum efficiency [125]. Other vanadate photocatalysts combining additional elements have been reported like InVO_4_ [126], Vanadium BiVO_4_ [127,128] wherein the BCC structure is maintained. Interestingly BiVO_4_ supports a number of crystal phases but only the monoclinic phase shows photocatalytic activity [129]. Bismuth vanadate photocataysts have also been reported for disinfection [130,131] with further enhancements in rate of disinfection reported with silver as a co-catalyst [58].

Other design led photocatalyst developments have concentrated on photocatalyst stability. Within the binary materials discussed above there is a general rule that occupancy of the d orbital determines stability with d^0^ and d^10^ materials remaining stable under reaction whilst all other materials exhibit photocorrosion [113]. This explains the difference in stability between Fe_2_O_3_ or ZnO (d^5^ and d^8^) and TiO_2_ or WO_3_ (d^0^) photocatalysts. However, the consequence of no d-orbital electron transitions often means photon excitation energies are generally large and in the UV domain and oxides of d^0^ or d^10^ elements have band gaps too large for efficient use of the solar spectrum [113]. To overcome this limitation oxynitride compounds of d^0^ materials, titanium oxynitrides and niobium oxynitrides, were developed. These materials have shown activity for disinfection with PdO nanoparticle modified TiON nanofibers reported for *E. coli* inactivation under visible light [132].

Two metal-layered oxides in perovskite structures have also been investigated [133]. Initial studies using d^0^ parents titanate and tantalite perovskites with a site occupancy of alkali metals produced stable catalysts, like SrTiO_3_, albeit with wider band gaps [134]. For a full review of the crystal structures (double, ternary, layered, *etc*. perovskites) band gaps produced from d^0^ metals with alkali metals and other light metals, the reader is referred to the paper by Eng *et al.* [135]. In the latter work the band gaps were measured in d^0^ compounds and it was found that in all crystal structures the band gap was largely determined by the central d^0^ metal ion. This considered, some researchers are utilizing d^0^ perovskite as photocatalysts under visible irradiation. Solid solution perovskites of d^0^ metals such as CeCo_0.05_Ti_0.95_O_3.97_ [136] or d^0^ metal compounds incorporating heavier ions such as Bi, Ag, In or Sn to produce AgNbO_3_ [137] NiNb_2_O_6_ NiTa_2_O_6_ [138] and other analogues have been reported as visible light activated photocatalysts. With the niobate materials such as Bi_2_O_2_CO_3_/Bi_3_NbO_7_ composites, K_4_Nb_6_O_17_ and Ag/Cu modified K_4_Nb_6_O_17_ reported for the inactivation of *E. coli* [139,140].

Organic semiconductors unbound to a surface represent homogeneous photocatalysts and are not the topic of this review and are not be used for disinfection due to issues associated with their recovery. However low dimensional organic semicondcutors like graphene oxide represent a new class of photocatalyst that can be separated from solution due to their large size in two dimensions. Graphene oxide, C_3_N_4_ and low dimensional photocatalysts have been reported for disinfection [141]. Due to the narrow band gaps they are normally supported on other materials as sensitizers to form semiconductor stacks a further new class of materials, produced by combining two existing photocatalysts.

Oxide semiconductor sensitized materials will be further discussed below. For example, tri-layer metal-free heterojunction photocatalysts based on RGO, α-S_8_ and CN sheets have been reported. Layered in different orders, namely CN-RGO-S_8_ and RGO-CN-S_8_, have been fabricated. Both of the photocatalytic structures have demonstrated bactericidal effect towards *E. coli* under visible-light irradiation with CN-RGO-S_8_ having higher inactivation rate than RGO-CN-S8 in aerobic conditions and *vice versa* [142]. Anatase TiO_2_ coated multiwalled carbon nanotubes (MWNT) demonstrated higher photocatalytic activity as compared to P25. TiO_2_ coated MWNT can inactivate *B. cereus* two times faster than P25. However, the same efficiency was not observed for *E. coli*, which could be due to the steric hindrance provided by the appendages of *E. coli* towards the 1-D nanotubes [143].

### 5.2. Modification and Sensitization of Existing Materials

Photocatalyst materials have been adapted for visible light activated disinfection by dye sensitized [144], plasmon sensitized [145] and semiconductor sensitized routes—*vide infra*. The mechanism of ROS generation in these materials is generally driven through oxygen reduction reactions by electron injected into the conduction band of the host semiconductor.

The addition of a second semiconductor material to an existing photocatalyst can provide a number of functions some simultaneously including; promoting charge carrier separation [146], acting as a charge carrier sink [147], acting as a co-catalyst to promote a specific reaction [148], and sensitization.

When a second semiconductor is used as a surface sensitizer the second material can be considered as an independent photocenter. In the example of *E. coli* disinfection [149] with CdS loaded TiO_2_, CdS can be excited independently of the parent photocatalyst injecting electrons in a manner similar to that of a dye sensitization. Surface sensitizers themselves are often nanostructured to alter their band-gap with the aim of increasing activity [150]. Some groups have extended this philosophy to consider molecular clusters of a few atoms as sensitizers [151]. These surface sensitizers often act in a manner similar to the action of the parent semiconductor with narrower band gap materials producing states above the valence band that can be observed via XPS while larger band gap materials did not produce VLA [152]. Another consideration when joining semiconductor materials and creating semiconductor junctions is the effect on the parent material. There is evidence that strongly polarizing species can affect the lattice structure raising the valence band in some materials [153].

### 5.3. Modification by Doping (Including Surface Loading)

Doping of photocatalysts to produce a visible light response is one of the most commonly reported approaches to improving the visible light activity. Matsunaga *et al*., in 1985, provided one of the earliest accounts of doped TiO_2_ for photoelectrochemical sterilization of microbial cells. Pt loaded TiO_2_ powders were demonstrated to inactivate *L. acidophilus*, *S. cerevisiae* and *E. coli* within 2 h [9]. Doping of ZnO nanostructures have been demonstrated to enhance the inactivation process either by shifting the band towards visible side or by dopant acting as co-catalyst. A number of dopants, including Pd [154], Ce [155], Co [156] and Ag [157], have been demonstrated to enhance the bacterial inactivation. C. Karunakaran *et al*., have investigated the effect of preparation method on photocatalytic inactivation activity of ZnO and Ag doped ZnO materials towards *E. coli*. The materials have been synthesized by three methods *i.e*., sol-gel [157] combustion [158] and microwave synthesis [159] out of which sol-gel synthesized materials have demonstrated the highest photocatalytic disinfection efficiency.

Metal ion dopants although the most studied for visible light activity there is a lack of consensus over efficacy, with as many reports claiming enhanced activity as a reduction in activity [160]. TiO_2_ doped with a variety of elements have been investigated for photocatalytic disinfection. Vohra *et al*., have explored Ag^+^ doped P25 TiO_2_ for disinfection of indoor air. *B. cereus*, *S. aureus*, *E.coli*, *A. niger*, and MS2 bacteriophage have been successfully inactivated, demonstrating high disinfection efficiency [161]. Ag doped TiO_2_ has also been effective in water disinfection [162]. Cu and S doped TiO_2_ nanoparticles have been effectively utilized for inactivation of *E. coli* and *M. lylae*, respectively [163,164]. Non-metals C, N, S, B, and the halogens as dopants for TiO_2_ and ZnO have been reviewed by Rehman *et al.* [165] and also by Im *et al.* [166]. Often co-doped materials are reported to have higher rates than the single dopant regimes as demonstrated by Li and co-workers, comparing nitrogen and carbon nitrogen co-doping on the inactivation rate for *E. coli* [167]. Second generation photocatalysts like WO_3_ [168] can also be doped to improve visible light activity, and present a further approach to developing new materials for disinfection [169].

## 6. Conclusions

In semiconductor photocatalysis, the primary reactions are electrochemical oxidation or reduction reactions involving hole and electron transfer from the photo-excited semiconductor. These redox reactions, in the presence of water and oxygen, can result in the production of reactive oxygen species (ROS), which can attack and inactivate microorganisms. The ROS include the hydroxyl radical (HO^•^), which has been suggested to be the primary species responsible for microorganism inactivation, however superoxide radical anion (O_2_^•−^), hydroperoxyl radical (HO_2_^•^) and hydrogen peroxide (H_2_O_2_) have been shown to contribute to the biocidal process. The ROS attack indiscriminately and therefore, emergence of antimicrobial resistance to photocatalysis is unlikely; however, a photocatalytic treatment must be adequate to avoid repair and regrowth of target organisms.

Around 750 million people are without access to an improved source for drinking and many more rely on sources that are unsafe. The development of a simple, yet inexpensive, water disinfection technology might help address the risk of waterborne disease in developing regions. Solar disinfection is recognized as an appropriate house-hold based treatment intervention and photocatalysis may be applied to enhance the solar disinfection efficiency; however, there are several challenges to be addressed before photocatalysis can be cheaply and efficiently deployed in developing regions.

In developing regions there is much concern about the risks of healthcare associated infections, which can result in death, increased bed stay, increased patient stress, and increased costs to the health service providers. One approach to reduce the risk of transmission of HCAI’s is to use “self-disinfecting” coatings on environmental surfaces within the healthcare setting e.g., bed rails, table tops, door handles. Photocatalytic coatings may be suitable for some surfaces; however, the coatings must be active for the inactivation of pathogenic microorganisms under ambient or indoor lighting conditions, and this poses a major challenge for the photocatalysis community. An important consideration is that the rate of photocatalytic disinfection is rather slow and the presence of organic contaminants will compete for ROS. Therefore, photocatalytic coatings for the disinfection of environmental surfaces under ambient lighting conditions should be additional to normal cleaning routines and may provide some residual surface disinfection to reduce the risk of transmission of infection. Another approach to reduce the incidence of HCAIs is to use photocatalytic coatings in the decontamination of medical devices including catheters, diagnostic tools and surgical instruments. The number of publications in the area of photocatalysis to address HCAIs is small but growing, and this is an important opportunity for researchers in the field. To assist in the development of efficient photocatalytic technology for solar water disinfection and the disinfection of surfaces in healthcare environments, novel materials are being explored which may be able to utilize visible light. Many of these materials have been investigated for other applications, mainly solar energy harvesting, but only a small number of these novel materials have been tested for their disinfection properties. Photocatalytic disinfection is a rapidly growing, challenging, and multi-disciplinary field, requiring the collaboration of researchers in microbiology and the physical sciences.
